# Urinary Bladder Invasive Squamous Cell Carcinoma in Juveniles

**DOI:** 10.24248/eahrj.v8i3.796

**Published:** 2025-01-30

**Authors:** Oscar Ottoman, Mohamed Muyeka, Edrick Elias, John Igenge, Magreth Magambo, Humphrey D Mazigo

**Affiliations:** a Department of Pathology, Bugando Medical Centre, Catholic University of Health and Allied Sciences, Mwanza, Tanzania; b Department of Urology, Bugando Medical Centre, Mwanza, Tanzania; c Department of Radiology, Bugando Medical Centre, Mwanza, Tanzania; d Department of Parasitology, Catholic University of Health and Allied Sciences, Tanzania.

## Abstract

**Background:**

Invasive squamous cell carcinoma of the urinary bladder caused by schistosomal infection is associated with aggressive complications and a poor prognosis. In schistosomiasis-endemic areas, it primarily affects adults over the age of 40 and rarely occurs in children under 15. For the first time at our hospital, we report a case of urinary bladder carcinoma associated with *Schistosoma haematobium* eggs in a 13-year-old child from northwestern Tanzania, a region endemic for *Schistosoma haematobium*.

**Case Presentation:**

A 13-year-old girl presented with left loin pain, turbid yellow urine, and upper limb pain for over a month. Multiple evaluations, including laboratory and ultrasonographic investigations, were conducted. Ultrasound findings revealed severe enlargement of both kidneys, with the left kidney being larger than the right. A computerized tomography (CT) scan showed severe bilateral hydronephrosis and hydroureter, likely due to vesicoureteral junction obstruction. A left nephrectomy was performed; however, the patient continued to experience dysuria. During cystoscopy, a tumor was identified on the left posterolateral wall of the bladder. Surgical exploration revealed adhesion of the tumor to the uterus, bladder neck, and cervix. A cystectomy was recommended, during which part of the right ureter was removed, and the remaining portion was anastomosed to the sigmoid colon. Histopathological examination of the tissue samples revealed invasive squamous cell carcinoma (Grade 1) involving the cervix and vaginal wall. Additionally, multiple active and calcified *Schistosoma haematobium* eggs were observed. The patient was referred to the oncology unit for radio-chemotherapy, where she continues to receive treatment.

**Conclusion:**

Chronic inflammatory responses associated with Schistosoma haematobium eggs in the urinary bladder walls can lead to severe complications affecting the entire urogenital system, regardless of age. These inflammatory responses may contribute to the development of squamous cell carcinoma even in young individuals.

## BACKGROUND

Bladder cancer is the most common malignancy of the urinary tract, accounting for approximately 77,000 new cases and 16,000 deaths annually in the USA.^1^ Worldwide, bladder cancer accounts for 1–3% of all malignancies ^2^. Transitional cell carcinoma (TCC), also referred to as urothelial carcinoma, is the predominant histological subtype, comprising 90–95% of bladder tumors. Other histological variants include squamous cell carcinoma (SCC), 2–5%) and other less common subtypes.^2^ In schistosomiasis-endemic regions of Africa, particularly in rural areas, the prevalence of SCC is notably higher than that of conventional TCC.^3,4^ Squamous cell carcinoma SCC of the bladder is classified into two subtypes: bilharzial-associated SCC (B-SCC), linked to schistosomiasis infection, and non-bilharzial-associated SCC (NBSCC), which occurs independently of schistosomiasis. These subtypes differ in epidemiology, natural history, and clinicopathological features.^3^ Bilharzial-associated SCC is the most common urothelial neoplasm in countries where schistosomiasis is endemic, and it demonstrates unique tumor characteristics.^5^ Schistosomiasis contributes to significant morbidity and mortality globally, with approximately 600 million people in tropical regions at risk of infection and 200 million currently affected.^6^ *Schistosoma haematobium* is the causative agent of urogenital schistosomiasis, which is prevalent in Sub-Saharan Africa and the Middle East.^7^ In endemic regions, schistosomiasis infection begins early in life, often before the age of five.^8^ Peak prevalence and infection intensity are observed between the ages of 10 and 20 years, followed by a decline due to behavioral changes, the development of natural immunity, and access to treatment.^8^ However, SCC associated with *Schistosoma haematobium* infection, including both B-SCC and NB-SCC, typically manifests later in life, between the ages of 50 and 70 years.^9^ In northwestern Tanzania, around Lake Victoria, data from the histopathology department at Bugando Medical Centre (BMC) revealed that SCC was the Urinary Bladder Invasive Squamous Cell Carcinoma www.eahealth.org most frequent histological type of bladder cancer (55.1%), with 44.9% of cases associated with schistosomal eggs. The mean age at diagnosis of urinary bladder SCC in this region was 54.3 years.^10^ *Schistosoma haematobium* induces chronic granulomatous inflammation, leading to bladder fibrosis, urinary stasis, and bacterial superinfection. Bacteria convert dietary nitrates and nitrites into nitrosamines, which are excreted in urine. These nitrosamines are carcinogenic, acting on the metaplastic epithelium and promoting the progression to squamous cell carcinoma.^11^

This report highlights a rare and incidental finding: the first documented case at our hospital of invasive urinary bladder carcinoma associated with *Schistosoma haematobium* eggs in a 13-year-old child from the Lake Victoria region, an endemic area for schistosomiasis. This case underscores the severe complications of invasive carcinoma on the genitourinary system in a patient during the first decade of life, a presentation not previously reported elsewhere.

### Case Presentation

A case of a 13-year-old female from western Tanzania presented as a self-referral from home with chief complaints of left loin pain, turbid yellow urine, and upper limb pain lasting for more than one month. Multiple evaluations were conducted to determine the cause, including laboratory and imaging investigations.

Laboratory investigations revealed the following findings:

**-Urinalysis:** Elevated white blood cells (WBC>100/HPF), hematuria (RBC>100/HPF), signs of active bacterial infection (positive nitrites and leukocytes +++).

**-Blood tests:** Increased uric acid level (290.00 µmol/L), elevated urea level (3.0 mmol/L), and increased serum creatinine level (64 µmol/L).

Ultrasonographic examination showed that both kidneys were severely enlarged, with the left kidney larger than the right. Based on these findings, the patient was diagnosed with severe bilateral hydronephrosis.

Further imaging with a CT scan confirmed severe hydronephrosis and hydroureter, likely due to vesicoureteric junction obstruction. The case was managed in accordance with the established protocol and surgical decision tree, leading to the recommendation and subsequent performance of a left nephrectomy. Tissue samples collected during the surgery were sent for histopathological evaluation, which confirmed chronic pyelonephritis with hydronephrosis.

**Figure 1.**
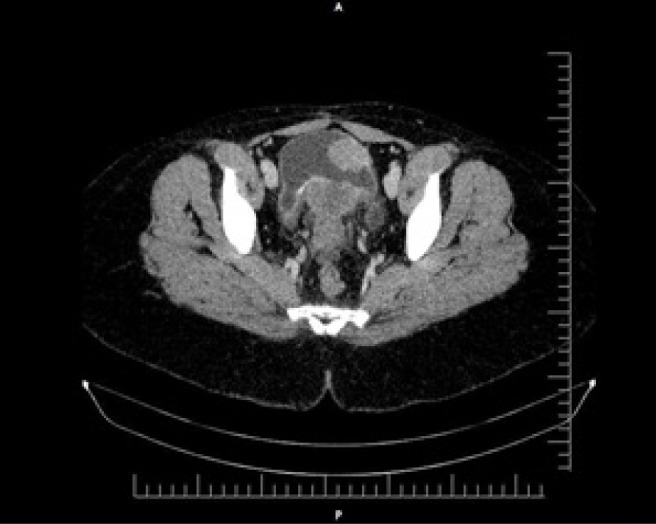
A Hypodense Mass Within the Retrovesical Space

Despite the nephrectomy, the patient continued to experience dysuria for a period of 10 months. A Kidney-Ureter-Bladder ultrasound was performed, which showed a normal urinary bladder with no visible lesions. However, the dysuria persisted. A pelvic CT scan was subsequently performed, revealing a hypodense mass within the retrovesical space displacing the calcified posterior wall of the bladder anteriorly. This finding was suggestive of chronic cystitis, possibly of schistosomal origin (Figure 1).

A Hypodense Mass Within the Retrovesical Space is displacing the calcified posterior wall of the bladder anteriorly, as indicated by the pink arrow. The calcified posterior bladder wall is suggestive of chronic cystitis, possibly of schistosomal origin

**Figure 2.**
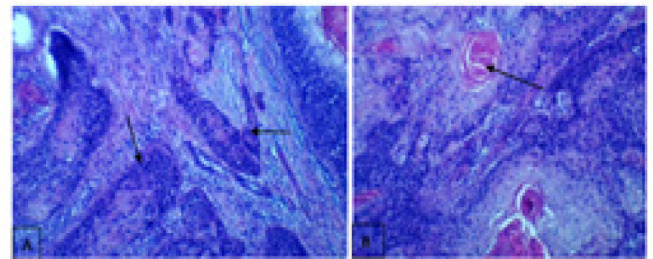
Invasive Trabecular and Neoplastic Trabecular Nests

**Figure 3.**
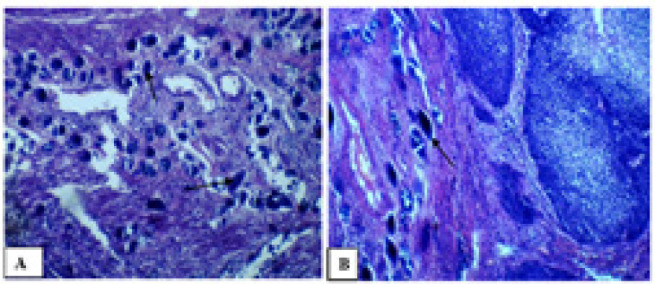
Calcified *Schistosoma Haematobium* Eggs and Calcified *Schistosomal* Eggs

The patient underwent a cystoscopy examination, which revealed the presence of a tumor on the left posterolateral wall of the bladder. Surgical intervention was performed, and intraoperative findings showed adhesion of the tumor to the uterus, bladder neck, and cervix. A cystectomy was carried out, during which part of the right ureter was removed and reimplanted into the sigmoid colon. Tissue samples were collected for histopathological examination. The report confirmed invasive squamous cell carcinoma (Grade I) with involvement of the cervix and vaginal wall (Figure 2). Additionally, multiple active and calcified *Schistosoma haematobium* eggs were identified within the detrusor muscle of the urinary bladder (Figure 3).

(A) Showing invasive trabecular nests of neoplastic polygonal-shaped cells with intracellular bridges, indicated by the black arrows on the H&E stain (x10 HPF). (B) Demonstrating the neoplastic trabecular nests of squamous cells with keratin pearls, indicated by the black arrow on H&E stain (x10 HPF).

(A) Showing multiple active and calcified *Schistosoma haematobium* eggs accumulated along the detrusor muscle of the urinary bladder, indicated by the black arrows on the H&E stain (x10 HPF). (B) Demonstrating the calcified *schistosomal* eggs accumulated near the trabecular nests of neoplastic squamous cells, indicated by the black arrow on H&E stain (x10 HPF).

During the last visit to the urology clinic, the patient still had a urinary diversion to the sigmoid colon and presented with back pain. A CT scan was recommended and performed, revealing a heterogeneous soft tissue mass measuring approximately 8.3 cm, which compressed the presacral space, along with a mass in the left iliac fossa. The conclusive diagnosis was metastatic disease and right hydroureteronephrosis. The patient was referred to the oncology unit for radio-chemotherapy treatment, but she attended only one visit and subsequently lost to follow-up.

#### Ethical Considerations

Permission for publication was granted by the CUHASBMC Joint Ethical Committee. Written informed consent was obtained from the parents for the publication of this case report and accompanying images.

## DISCUSSION

In most endemic areas with a high prevalence of urogenital schistosomiasis, particularly in Sub-Saharan Africa, an increase in urinary bladder cancer has been reported.^12^ Most cancers associated with *Schistosoma* infections have uncharacteristic features that depend on the stage of diagnosis, patient age, and squamous cell carcinoma (SCC) subtype.^13^ More than 90% of bladder cancer cases associated with *S. haematobium* are urothelial carcinomas, which is in contrast to the patterns observed in Western countries and non-endemic areas.^12,14^

Our case highlights invasive squamous cell carcinoma associated with *S. haematobium* infection in a 13-year-old, which is quite different from the data reported in the northwestern region of Tanzania around Lake Victoria. In this region, bladder cancer typically occurs in adults, with the mean age at diagnosis of urinary bladder SCC being 54.3 years.^10^

*Schistosoma haematobium* is a common parasite contributing to bladder cancer risk. The underlying mechanism involves chronic granulomatous inflammation, which leads to bladder fibrosis, urinary stasis, and bacterial superinfection. Bacteria can convert dietary nitrates and nitrites into nitrosamines, which are excreted in urine.^11^ These nitrosamines are carcinogenic and act on metaplastic epithelium, ultimately leading to squamous cell carcinoma.^11^

In the urinary bladder, chronic inflammatory reactions caused by *Schistosoma* eggs alter the immune system, increasing susceptibility to co-infections with other bacteria and viruses.^15,16^ This can contribute to the early onset of disease.^17^ Similarly, in our case, recurrent infections likely contributed to bladder carcinoma, exacerbated by living in an endemic area with a high frequency of *Schistosoma* infestation.^17^

In endemic areas, individuals may present with mild symptoms, while repeated infections can result in complications such as obstructive uropathy with hydronephrosis, hypertension, liver fibrosis, esophageal varices, or squamous cell carcinoma and urothelial carcinoma of the bladder. Renal involvement, commonly seen with *S. haematobium* infection, results from fibrosis and calcification of tissue-trapped ova in the lower urinary tract, causing obstruction, reflux, infection, and stone formation. This process can lead to interstitial nephritis, progressing to end-stage renal disease.^18,19,20^

Our case presented with similar complications. Diagnosis typically relies on cystoscopy, biopsy, and careful bimanual examination under anaesthesia.^18^ In our case, a EUA (examination under anaesthesia), cystoscopy, and biopsy provided a definitive histopathological diagnosis. Urine cytology is also a valuable diagnostic tool for SCC in bilharzial patients,^21^ and cytokeratin shedding in urine has been used as a biomarker for early SCC detection, particularly in screening programs.^21^

Radical cystectomy remains the treatment of choice. In males, this includes excision of the bladder, prostate, and perivesical fat. In females, it involves anterior pelvic exenteration, which includes the removal of the bladder, perivesical fat and peritoneal coverage, urethra, uterus, ovaries, and anterior vaginal wall.^22,23^ However, the five-year survival rate for this approach ranges from 34% to 50%.^24^ In our case, a cystectomy, left nephrectomy, hysterectomy, and salpingectomy were performed, along with urinary diversion.

Bilharzial bladder squamous cell carcinoma serves as an example of a preventable malignant disease.^25^ Primary prevention is achievable through the nationwide elimination of the parasite, combining snail control with mass treatment of infested rural populations using oral antibilharzial drugs.^25^ Secondary prevention is also possible through early detection of the disease in rural populations via urine cytology.^25^ Selective screening of high-risk groups, especially populations living near Lake Victoria, has proven effective for early detection, with a yield of two cases per 1000 individuals screened in endemic areas.^25^

The low accessibility of screening and antibilharzial medication among populations in endemic areas likely explains the occurrence of invasive bilharzial bladder SCC in juveniles in our region.

## CONCLUSION

Chronic infections caused by *Schistosoma haematobium* can lead to severe complications, including cancer, chronic renal failure due to hydronephrosis and urine flow obstruction, as well as other extra-urinary manifestations. Bladder cancer associated with schistosomal infections is often characterized by the squamous cell carcinoma subtype, even in young individuals. This case report highlights the critical need for repeated mass drug administration with praziquantel in endemic areas to combat *S. haematobium* infections. Such interventions can significantly reduce the risk of developing severe and irreversible morbidities, including urinary bladder carcinomas.
